# Non-Lead Protective Aprons for the Protection of Interventional Radiology Physicians from Radiation Exposure in Clinical Settings: An Initial Study

**DOI:** 10.3390/diagnostics11091613

**Published:** 2021-09-03

**Authors:** Mamoru Kato, Koichi Chida, Masato Munehisa, Tadaya Sato, Yohei Inaba, Masatoshi Suzuki, Masayuki Zuguchi

**Affiliations:** 1 Course of Radiological Technology, Health Sciences, Tohoku University Graduate School of Medicine, 2-1 Seiryo, Aoba-ku, Sendai 980-8575, Japan; kato-amc@med.tohoku.ac.jp (M.K.); inaba@med.tohoku.ac.jp (Y.I.); msuzuki@irides.tohoku.ac.jp (M.S.); qqrm6wq9k@arrow.ocn.ne.jp (M.Z.); 2 Akita Cerebrospinal and Cardiovascular Center (Akita Medical Center), 6–10 Senshu-Kubota Machi, Akita 010-0874, Japan; masatom1025@gmail.com (M.M.); tsatotan@gmail.com (T.S.); 3 Department of Radiation Disaster Medicine, International Research Institute of Disaster Science, Tohoku University, 468-1 Aramaki Aza-Aoba, Aoba-ku, Sendai 980-0845, Japan; 4 Department of Cardiovascular Medicine, Senseki Hospital, 53-7 Akai, Aza Dai, Higashi Matsushima 981-0501, Japan; 5 Department of Cardiovascular Medicine, Saka General Hospital, 16-5 Nishiki-machi, Shiogama 985-8506, Japan

**Keywords:** radiation protection, fluoroscopy, interventional radiology (IVR), fluoroscopically guided interventional procedures, percutaneous coronary intervention (PCI), protective apron, radiation safety, radiation dose, X-ray examination, disaster medicine

## Abstract

Radiation protection/evaluation during interventional radiology (IVR) poses a very important problem. Although IVR physicians should wear protective aprons, the IVR physician may not tolerate wearing one for long procedures because protective aprons are generally heavy. In fact, orthopedic problems are increasingly reported in IVR physicians due to the strain of wearing heavy protective aprons during IVR. In recent years, non-Pb protective aprons (lighter weight, composite materials) have been developed. Although non-Pb protective aprons are more expensive than Pb protective aprons, the former aprons weigh less. However, whether the protective performance of non-Pb aprons is sufficient in the IVR clinical setting is unclear. This study compared the ability of non-Pb and Pb protective aprons (0.25- and 0.35-mm Pb-equivalents) to protect physicians from scatter radiation in a clinical setting (IVR, cardiac catheterizations, including percutaneous coronary intervention) using an electric personal dosimeter (EPD). For radiation measurements, physicians wore EPDs: One inside a personal protective apron at the chest, and one outside a personal protective apron at the chest. Physician comfort levels in each apron during procedures were also evaluated. As a result, performance (both the shielding effect (98.5%) and comfort (good)) of the non-Pb 0.35-mm-Pb-equivalent protective apron was good in the clinical setting. The radiation-shielding effects of the non-Pb 0.35-mm and Pb 0.35-mm-Pb-equivalent protective aprons were very similar. Therefore, non-Pb 0.35-mm Pb-equivalent protective aprons may be more suitable for providing radiation protection for IVR physicians because the shielding effect and comfort are both good in the clinical IVR setting. As non-Pb protective aprons are nontoxic and weigh less than Pb protective aprons, non-Pb protective aprons will be the preferred type for radiation protection of IVR staff, especially physicians.

## 1. Introduction

The occupational exposure and patient radiation dose are important issues [1,2,3,4,5]. Especially, interventional radiology (IVR) procedures deliver high radiation doses to both the physician and patient [6,7,8,9,10,11,12,13,14]. Furthermore, IVR physicians are at high risk of radiation-induced injury [15,16,17,18,19,20]. Thus, radiation evaluation/protection is very important to the IVR physician [21,22,23,24,25].

Although physicians should wear protective aprons, the IVR physician may not tolerate wearing one for long procedures because protective aprons are generally heavy. In fact, orthopedic problems are increasingly reported in IVR physicians due to the strain of wearing heavy aprons during IVR [26,27,28].

Recently, non-lead (Pb) protective aprons (lighter weight, composite materials) have been developed [29,30]. Although non-Pb protective aprons are more expensive than Pb protective aprons, the former protective aprons weigh less. Furthermore, non-Pb protective aprons are environmentally friendly. Pb toxicity is obviously not in play. Thus, non-Pb protective aprons are optimal. However, whether the protective performance of non-Pb aprons is sufficient in the IVR clinical setting is unclear. Furthermore, the optimal Pb-equivalent of protective aprons, 0.25- and 0.35-mm Pb-equivalents, is unclear in the IVR clinical setting.

This study compared the ability of non-Pb and Pb protective aprons (0.25- and 0.35-mm Pb-equivalents) to protect physicians from scatter radiation in a clinical setting (cardiac catheterizations, including percutaneous coronary intervention, PCI) using an electric personal dosimeter (EPD). Physician comfort levels in each apron during procedures were also evaluated.

The purpose of this initial study was to demonstrate the effectiveness of non-lead protective aprons for the protection of IVR physicians from radiation exposure in clinical settings.

## 2. Materials and Methods

### 2.1. Protective Apron and Radiation Measurement

In this study, physicians wore one of four types of protective apron at random: 0.25-mm Pb (QA 0.25, Kasei-optonics, Odawara, Japan), 0.35-mm Pb (QA 0.35, Kasei-optonics, Odawara, Japan), non-Pb, 0.25-mm Pb-equivalent (HGA 0.25, Kasei-optonics, Odawara, Japan), or non-Pb, 0.35-mm Pb-equivalent (HGA 0.35, Kasei-optonics, Odawara, Japan). Pb protective aprons consist of lead. Non-lead protective aprons consist of composite materials, mainly tungsten (W) and tin (Sn).

**Figure 1:**
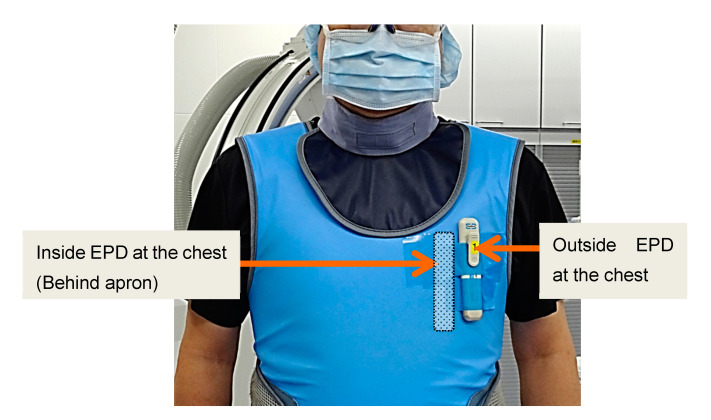
The position of the two electric personal dosimeters (EPDs) worn on the physician’s protective apron in a clinical setting.

For radiation measurements, physicians wore two EPDs (PDM-117, Hitachi-Aloka, Taito-ku, Japan): One inside a personal protective apron at the chest, and one outside a personal protective apron at the chest. Figure 1 indicates the position of each EPD in the clinical setting (cardiac catheterizations, including PCI). We evaluated external (average dose of exterior EPDs at the chest) and internal doses (average dose of interior EPDs at the chest).

The radiation shielding effects (%) of the protective aprons were also determined as follows:

Radiation shielding effects (%) = (1 − inside dose/outside dose) × 100.

### 2.2. Cardiac Catheterization

Radiation exposure (external and internal radiation dose) for the two physicians during more than 50 cardiac catheterizations (including PCI) with the four types of protective apron were measured randomly at Akita Medical Center (Akita, Akita, Japan) (Table 1). We did not establish inclusion or exclusion criteria for this initial study. During each procedure, physicians were at liberty to choose any of the four types of protective apron.

**Figure 2:**
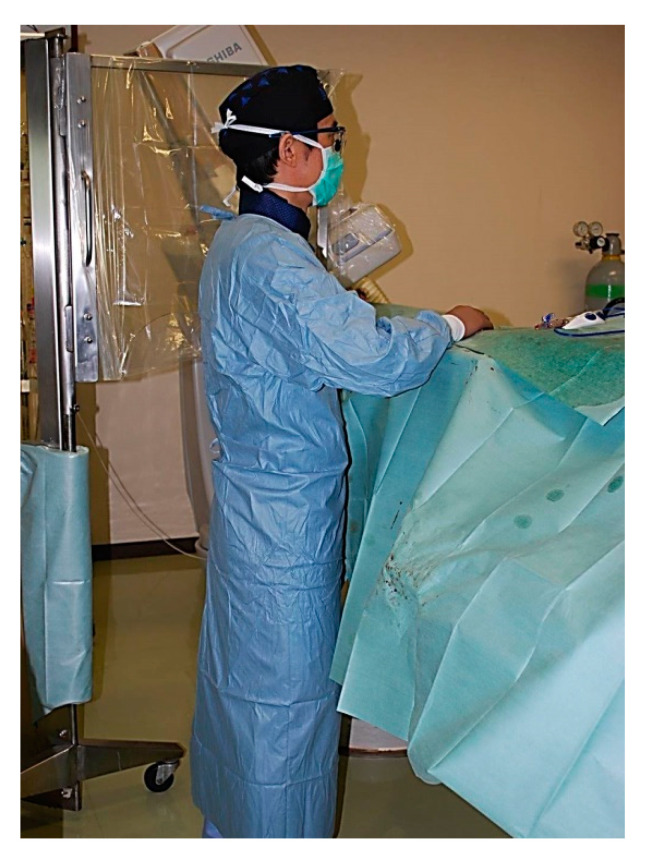
Photograph during the cardiac catheterization procedure.

In this study, an additional lead acrylic protection device was also used, if possible, during procedures (Figure 2).

Physician comfort levels in each protective apron during cardiac catheterizations procedures (including PCI) were also determined through interviews.

The procedures (cardiac catheterizations, including PCI) were performed using a digital cine X-ray single-plane system (Infinix Celeve-i, Toshiba, Ohtawara, Japan) with a 7-inch mode flat-panel detector, an acquisition (cine) rate of 15 frames/s, and pulsed fluoroscopy (15 pulses/s).

This study was approved by the Ethics Committee of Akita Cerebrospinal and Cardiovascular Center (Akita Medical Center). We also evaluated the radiation dose indicator (cumulative air karma [AK]) and fluoroscopy time undergoing cardiac catheterizations, including PCI.

## 3. Results

The X-ray procedure details used in cardiac catheterizations (including PCI) are shown in Table 1. Table 2 summarizes the findings of this clinical study. Although the radiation-shielding effect of the 0.35-mm Pb protective apron was the best (98.9%) among the four types, physician comfort was the worst (very poor) because this protective apron was the heaviest. Conversely, although physician comfort in the non-Pb 0.25-mm Pb-equivalent protective apron was highest (excellent) among the types because the protective apron was the lightest, the radiation shielding effect was the worst (96.1%).

The performance (both the shielding effect (98.5%) and comfort (good)) of the non-Pb 0.35-mm-Pb-equivalent protective apron was good in the clinical setting. The radiation-shielding effects of the 0.35-mm Pb and non-Pb 0.35-mm-Pb-equivalent protective aprons were very similar. The extent of physician comfort when wearing the non-Pb 0.35-mm-Pb-equivalent protective apron was similar to that when wearing the non-Pb 0.25-mm-Pb-equivalent protective apron. Thus, non-Pb 0.35-mm Pb-equivalent protective aprons may be more suitable in providing radiation protection for IVR physicians. Thus, we recommend that IVR physicians should wear the non-Pb 0.35-mm-Pb-equivalent protective apron.

## 4. Discussion

In X-ray examination, radiation protection/evaluation of physicians and patients is significant. Although the wide acceptance of IVR procedures, such as PCI, has led to increasing numbers of interventions being performed, radiation exposures from IVR are conclusively higher, exposing both the IVR staff and the patient to high radiation doses. [31,32,33,34,35,36,37,38,39].

Many radiation-related injuries caused by excessive radiation exposure during cardiac intervention IVR procedures have been reported [40,41,42,43]. Therefore, radiation monitoring for physicians is essential in reducing the radiation injury risk during IVR. Furthermore, most IVR physicians stand close to the patient where the scattered radiation and consequently the physicians’ exposure is higher. Therefore, radiation protection/measurement for the physician during IVR poses a very important problem [44,45,46,47,48,49,50,51,52,53,54].

A protective apron is inevitably heavy but should be worn by all staff working in catheterization suites. The protective aprons increase the risk of musculoskeletal disorders. Careful selection of a personal protective apron is thus important [55,56].

As non-lead aprons consist of composite materials, mainly W and Sn, they are approximately 20% lighter than lead aprons [29,30]. In the phantom study, the performance of these non-Pb and Pb protective aprons was similar for scattered X-rays [30]. However, whether the performance (both shielding effect and comfort) of non-Pb aprons is sufficient in the clinical setting (cardiac catheterizations including PCI) is unclear. In this study, the radiation protection provided by non-Pb and Pb protective aprons in clinical IVR settings are compared. As a result, we demonstrated the effectiveness of non-Pb protective aprons for the protection of IVR physicians from radiation exposure in clinical IVR settings.

Namely, our results showed that the performance (both shielding effect and comfort) of the non-Pb 0.35-mm Pb-equivalent protective apron was good in the clinical setting (cardiac catheterizations including PCI). Thus, non-Pb 0.35-mm Pb-equivalent protective aprons may be more suitable in providing radiation protection for IVR physicians.

For procedures during which non-lead protective aprons were worn, the mean external dose tended to be lower than that when lead protective aprons were worn (non-lead protective aprons 142.6 or 123.9 μSv; lead protective aprons 166.4 or 209.2 μSv, Table 2). Moreover, the cumulative AK was higher (non-lead protective aprons 1345.7 or 1345.3 mGy, lead protective aprons 1037.2 or 812.3 mGy, Table 1) and the fluoroscopy time longer (non-lead protective aprons 34.5 or 31.7 min, lead aprons 30.4 or 21.2 min, Table 1). The probable explanation is that an additional, lead-containing, acrylic protection device was employed during many procedures featuring non-lead protective aprons. Thus, if the cumulative AK was higher, the external dose was lower because the non-apron device shielded staff from scattered radiation. We did not evaluate the effect of the non-apron device because the shielding effect (%) is relative. 

Possible ergonomic improvements include the use of a two-part protective apron (that separately protects the chest and waist). This would distribute the protective apron weight more equally across the shoulders and waist, possibly reducing the risk of musculoskeletal pain [56].

### Limitation

This was an initial study of non-Pb protective apron use in clinical settings. A controlled comparison of four groups (wearing non-Pb and Pb protective aprons; 0.25- and 0.35-mm-Pb-equivalents), with statistical evaluation, is necessary.

## 5. Conclusions

This study compared the protective performance of Pb and non-Pb aprons of 0.25-mm and 0.35-mm Pb-equivalents in the clinical setting (cardiac catheterizations including PCI). Non-Pb 0.35-mm Pb-equivalent protective aprons may be more suitable for providing radiation protection for IVR physicians because the shielding effect and comfort are both good in the clinical setting.

As non-Pb protective aprons are nontoxic and weigh less than Pb protective aprons, and IVR staff mainly receive doses from scattered X-rays, non-Pb protective aprons will be the preferred type for radiation protection of IVR staff, especially physicians.

## Figures and Tables

**Table 1:** A summary of our study.

Protective Apron	Number of Procedures			Cumulative AK	Fluoroscopy Time
Pb-Equivalent [mm]	Total	CAG	PCI	[Mean ± SD, mGy]	[Mean ± SD, min]
Non-lead	78	32	46	1345.7 ± 1378.9	34.5 ± 27.7
0.25					
Non-lead	51	19	27	1345.3 ± 1637.3	31.7 ± 27.1
0.35					
Lead	50	23	27	1037.2 ± 824.7	30.4 ± 21.7
0.25					
Lead	50	26	24	812.3 ± 704.9	21.2 ± 15.5
0.35					

AK: Air karma, CAG: Coronary angiography, PCI: Percutaneous coronary intervention.

**Table 2:** Summary of our study on the performance of protective aprons in the clinical setting. Each was used for more than 50 catheterizations, including PCI.

Protective Apron	Apron Weight	Outside (External) Dose	Inside (Internal) Dose	Shielding Effects	Comfortably
Pb-Equivalent [mm]	[kg]	[Mean ± SD, μSv]	[Mean ± SD, μSv]	[%]	
Non-lead	1.8	142.6 ± 199.3	5.5 ± 7.1	96.1	Excellent
0.25					
Non-lead	2.9	123.9 ± 99.3	1.8 ± 1.5	98.5	Good
0.35					
Lead	3.0	166.4 ± 153.6	4.6 ± 4.7	97.3	Somewhat poor
0.25					
Lead	3.8	209.2 ± 163.1	2.2 ± 1.9	98.9	Very poor
0.35					
